# Genetic Variation and Combining Ability Analysis of Bruising Sensitivity in *Agaricus bisporus*

**DOI:** 10.1371/journal.pone.0076826

**Published:** 2013-10-08

**Authors:** Wei Gao, Johan J. P. Baars, Oene Dolstra, Richard G. F. Visser, Anton S. M. Sonnenberg

**Affiliations:** 1 Plant Breeding, Wageningen University and Research Center, Wageningen, The Netherlands; 2 Institute of Agricultural Resources and Regional Planning, Chinese Academy of Agricultural Sciences, Beijing, PR China; Kyushu Institute of Technology, Japan

## Abstract

Advanced button mushroom cultivars that are less sensitive to mechanical bruising are required by the mushroom industry, where automated harvesting still cannot be used for the fresh mushroom market. The genetic variation in bruising sensitivity (BS) of *Agaricus bisporus* was studied through an incomplete set of diallel crosses to get insight in the heritability of BS and the combining ability of the parental lines used and, in this way, to estimate their breeding value. To this end nineteen homokaryotic lines recovered from wild strains and cultivars were inter-crossed in a diallel scheme. Fifty-one successful hybrids were grown under controlled conditions, and the BS of these hybrids was assessed. BS was shown to be a trait with a very high heritability. The results also showed that brown hybrids were generally less sensitive to bruising than white hybrids. The diallel scheme allowed to estimate the general combining ability (GCA) for each homokaryotic parental line and to estimate the specific combining ability (SCA) of each hybrid. The line with the lowest GCA is seen as the most attractive donor for improving resistance to bruising. The line gave rise to hybrids sensitive to bruising having the highest GCA value. The highest negative SCA possibly indicates heterosis effects for resistance to bruising. This study provides a foundation for estimating breeding value of parental lines to further study the genetic factors underlying bruising sensitivity and other quality-related traits, and to select potential parental lines for further heterosis breeding. The approach of studying combining ability in a diallel scheme was used for the first time in button mushroom breeding.

## Introduction

*Agaricus bisporus* (button mushroom) is one of the most widely cultivated mushrooms in the world. The Netherlands is one of the largest button mushroom producers (230,000 tons in 2009) [1], and mechanical harvesting is widely used in the Dutch mushroom industry in order to reduce the high labor costs. So far, mechanical harvesting is mostly used for industrially processed mushrooms and not for the fresh market. Mushrooms for the fresh market are now handpicked and this represents a large cost factor. However, mechanical harvesting causes bruising and discoloration leading to a lower mushroom quality, a shorter shelf life and thus a lower price. Fully automated harvesting of button mushrooms for the fresh market requires strains that are less sensitive to mechanical damage. In order to develop such varieties, a better understanding of the genetic basis for cap discoloration after mechanical damage is needed. Mushroom discoloration caused by mechanical damage is a consequence of enzyme-catalyzed oxidation of phenols into quinones. These slightly colored quinones undergo further reactions forming dark melanins [2,3]. It is assumed that the enzymes and substrates are physically separated in different cellular compartments. Visible-near-infrared spectroscopy was used to confirm the release of vesicular contents during cytoplasm breakdown and the activation of tyrosinase which catalyzes the oxidation of phenolics to quinones [4]. Several factors can thus affect discoloration after bruising, e.g. substrates, enzymes and intracellular membranes. Genetic analysis for bruising sensitivity is a tool for unraveling the basis of different mechanisms involved in mushroom bruising and discoloration, which is the prerequisite for mushroom breeding.

Mushroom breeding with homokaryons shows striking similarities with hybrid maize breeding making use of inbred lines. The combining ability analysis approach worked out in maize by Sprague and Tatum (1942) is of direct use to determine the breeding value of parental lines [5]. This approach allows separation of the combining ability among lines into a general and a specific part, usually referred to as general and specific combining ability (GCA and SCA, respectively). The first refers to the mean contribution of lines to the performance of hybrids, and the latter refers to deviation from the expected hybrid performance using GCA estimates of the parental lines. Different methods have been used to estimate combining ability of lines [5]. The GCA of a line is commonly used in plant breeding as a measure of its breeding value. The GCA of inbred lines is determined and used in maize breeding to predict promising hybrids [6,7]. This approach was also successfully applied in the development of sunflower hybrids with improved resistance to *Phomopsis* [8]. Interesting combining ability analyses were recently performed in watermelon [9] and oil palm [10]. A GCA estimate is considered to be a good indicator of the relative value of a parental line in terms of frequency of favorable genes and of its genetic divergence, which allows the selection of superior parental lines. The differences between lines in GCA are mainly due to the additive and additive × additive gene interactions, whereas the differences in the SCA of lines are attributable to non-additive, often dominant epistatic interactions [8]. GCA and SCA estimates can depend strongly on environmental factors as shown for grain yield in maize [11,12]. The combining ability of lines usually has a polygenic base and is trait-dependent as shown in a study in rice to elucidate heterosis for ten agronomic traits [13]. The estimates for GCA and SCA facilitate heterosis breeding and enable a dedicated choice of parents to create segregating populations for genetic analysis.

The method of diallel crosses was first applied to fungi by Simchen and Jinks (1964) in *Schizophyllum commune*, where the variation in mycelium growth rate within a set of crosses between monokaryons was studied. The variation was shown to be due to both additive and dominant gene action [14]. Later on, this method was used in genetic and breeding studies in this species [15–17]. The analysis of combining ability was used also in genetic studies of growth rate and mushroom production in oyster mushroom, *Pleurotus sapidus*. In that study, a random monokaryon was selected for each of the eight wild dikaryons, and the eight monokaryons were crossed in a diallel scheme [18].

Breeding value can also be estimated in button mushroom (*A. bisporus*)*. A. bisporus* is a member of the Homobasidiomycetes. Homobasidiomycets are characterized by the fact that they contain two types of haploid nuclei with different mating types that stay apart in each cell. Fusion of nuclei only takes place in basidial cells just before spores are produced. Each diploid nucleus produces four haploid nuclei after meiosis and these are distributed to the four spores formed by each basidial cell. The spores germinate into haploid mycelium that cannot produce fruiting bodies. These infertile mycelia are designated as homokaryons. Homokaryons with different mating type can anastomose and subsequent exchange of nuclei leads to the formation of heterokaryotic (dikaryotic) mycelium. The presence of both mating types within one mycelial cell triggers a developmental process leading to the formation of fruiting bodies provided environmental conditions are favorable. This non-self compatibility or heterothallism is controlled by one or two unlinked loci. The outbreeding potentials of basidiomycetes are high because they possess numerous distinct mating types [19]. The majority of Homobasidiomycetes show this heterothallic life cycle. The button mushroom *A. bisporus* deviates from this life cycle. Most basidia produce only two spores and the four post-meiotic nuclei are distributed over two spores in such a way that non-sister nuclei are paired in one spore [20,21]. This usually leads to mycelia with two different mating types and thus to fertile heterokaryons. This type of life cycle is designated as secondary homothallic. This phenomenon is also referred to as automixis or intra-tetrad mating, a form of selfing where mating occurs among the products of a single meiosis. It is rare that basidia produce three or four spores. Only on these basidia spores are produced with one haploid nucleus that generate homokaryons and can be used for cross breeding. Two decades ago, a novel variety has been found in de Sonoran desert of California [22]. This variety produces predominantly four spored basidia and each spore germinates into homokaryotic mycelia. The two varieties are designated as *A. bisporus* var. *bisporus* and *A. bisporus* var. *burnetti*, respectively. Since var. *burnetti* is poor in various agronomic and quality traits, all commercially cultivated strains are *Agaricus bisporus* var. *bisporus*. The ability of mating homokaryons with different mating types enables the evaluation of the general performance of a particular homokaryon under different genetic backgrounds and the estimation of the breeding value for the homokaryon in mushroom breeding programs. Homokaryotic lines with contrasting performance are commonly used to generate segregating populations and map genomic regions involved in traits and identify candidate genes [23].

A collection of wild strains and traditional cultivars of the button mushroom was screened for bruising sensitivity (BS). The results indicated that some wild strains showed less BS than commercial lines [24]. A very common activity in breeding is the transfer of a new desirable trait derived from a wild line (donor strain) into existing commercial lines (recipient strains) through recurrent backcross selection [25]. In this study, the homokaryotic parental lines of some selected heterokaryotic lines were recovered through protoplasting. These lines were used to generate a diallel set of crosses in order to study the combining ability for BS and the breeding value of the parental lines. This has never been done before in button mushroom breeding. This study intends to examine whether this approach provides a good basis for the selection of parental lines for breeding advanced cultivars that are less sensitive to mechanical bruising and the choice of parental lines for genetic studies to elucidate genes involved in BS and other agronomic traits.

## Materials and Methods

### Selection of heterokaryotic parental strains

A broad selection of varieties from the culture collection of Wageningen UR-Plant Breeding was recently studied for sensitivity to bruising [24]. The selection included wild isolates originating from different geographic regions, traditional strains generated by single spore or multi-spore selection that were used before 1980, and present-day hybrids used worldwide since 1981. Strain CH 1, CH2, TW6, TW7 and TW8 are known to be genetically related from previous studies. These strains showed a large variation in bruising sensitivity and cap color. Eighteen bruising resistant and sensitive strains were selected (Table 1) and used to recover the constituent homokaryotic parental lines by protoplasting the vegetative mycelium.

**Table 1:** Bruising resistant and sensitive strains used to generate homokaryons.

No.	CODE	sensitivity	color	No.	CODE	sensitivity	color
1	WB18	resistant	brown	10	TW8	resistant	white
2	WB2	resistant	brown	11	WW2	resistant	white
3	WB4	resistant	brown	12	WB15	sensitive	brown
4	WB5	resistant	brown	13	WB16	sensitive	brown
5	WW1	resistant	off-white	14	WB17	sensitive	brown
6	CH1	resistant	white	15	TW6	sensitive	white
7	CH2	resistant	white	16	TW7	sensitive	white
8	TO7	resistant	white	17	WW7	sensitive	white
9	TW1	resistant	white	18	WW8	sensitive	white

TW = traditional white; CH = commercial hybrid; TO = traditional off-white; WW = wild white; WB = wild brown.

### Protoplasting and protoclone isolation

Vegetative mycelium of 18 selected strains was protoplasted according to the method described by Sonnenberg et al. (1988). Suspensions containing protoplasts were serially diluted and plated onto MMP (1% malt extract, 0.5% mycological peptone, 10 mM MOPS, and 1.75% agar, pH7.0) + 0.6 M Sucrose for regeneration. After incubation at 24 °C for 5-7 days around 200 regenerated single protoplasts (protoclones) were isolated for each strain under a stereo microscope and transferred to a new MMP plate. Protoclones were sub-cultured onto MMP plates covered with cellophane. After 10-14 days incubation at 24 °C, the mycelium from the cellophane was put into a micro-centrifuge tube for DNA extraction.

### Identification of homokaryons

Initial selection of putative homokaryons was based on colony morphology and growth rate that differed from the original heterokaryons. Homokaryons were identified using PCR methods. Primers (Table 2) designed based on known gene sequences were tested for their ability to screen putative homokaryotic protoclones. The absence of one band compared to that of heterokaryons was taken as an indication that a particular protoclone was homokaryotic. Alternatively, ISSR–PCR was used. Each PCR (15µL) with primers G-6-PD, 39Tr 2/5-2/4, PIN150 and P33N10 contained 20 ng DNA template, 1× incubation buffer, 300µM each dNTP, 15 pmol primer, 0.3U Taq DNA polymerase; each PCR (15µL) with ISSR primers contained 2 ng DNA template, 1× incubation buffer, 300µM each dNTP, 2 pmol primer, 0.3 U Taq DNA polymerase. Amplifications were performed as follows: after an initial denaturing step at 94 °C for 5 min, the samples were processed through 35 cycles, each consisting of 45 s at 94 °C, 45 s at annealing temperature 58 or 55 °C and 90 s at 72 °C, and a final extension step at 72 °C during 5 min. PCR products were separated on 1% agarose gels. The genetic diversity present in the set of recovered parental lines was studied with the KASPar SNP genotyping system (KBiosciences Competitive Allele Specific PCR SNP genotyping system) using 535 SNP markers. The genotyping was done by Dr. Van Haeringen Laboratorium B.V., Wageningen. These markers showed sequence polymorphisms between CH2B (http://genome.jgi-psf.org/Agabi_varbisH97_2/Agabi_varbisH97_2.home.html) and CH2A (resequenced by ServiceXs, Leiden, the Netherlands). The similarity (Squared Euclidean distance) between strains was calculated with SPSS (IBM, 19th edition) with the method of between-group linkage. A dendrogram was made to present the degree of similarity between the recovered homokaryotic lines.

**Table 2:** Molecular markers used for identification of homokaryons.

Marker		Sequence (5’–3’)	Ta (°C)	Polymorphic Strains	Reference
G-6-PD	Forward	GTAATGTACACGGAGAC	58	TW8, WB2,TW7, WW2	This article
	Reverse	ACTCTGAAGGAACTTGG			
39Tr 2/5-2/4	Forward	CCTCGCGCAAGCAGATACAA	58	TW1, WB5, TW5	This article
	Reverse	TTGTCCGAGACTTACTCACG			
PIN 150	Forward	AGGTGACATGTCAGAAGCGC	58	CH1, CH2, TW6	[34,35]
	Reverse	CAATCTCAAGCTTGCCTGG			
P33N10	Forward	ACTATAAAGCGTGAGCTATACG	58	WW7	[34,35]
	Reverse	TATCTTCTGCGCTGTGTTGCT			
ISSR A2		HVV(GTT)5	55	TO7, WW1, WB18	This article
ISSR A7		VVH(TTG)5	55	WB4	This article
ISSR B		NDV(CT)8	55	WW8, WB16	This article
ISSR A		NDB(CA)7-C	55	WB17	This article

Ta: annealing temperature

### Crossing between homokaryons

Homokaryons obtained through protoplasting were inter-crossed on compost agar plates (75 g dried milled phase II compost of 0.1 mm particle size was suspended in 1 L of tap water together with 17.5 g of agar. The medium was sterilized for 1 hour at 121°C and poured into petri dishes). Crossings were identified based on the morphology of mycelium interactions and confirmed using PCR. Mycelium of the contact zone was isolated and transferred onto a new MMP medium plate to confirm that it was homogenous, after which the cross was confirmed with the same PCR primers that were used for homokaryon identification.

### Fruiting test

Crossings that were identified as hybrids were put in a climate controlled fruiting test. Hybrid strains were grown in trays (56 × 36 ×20 cm) filled with 16 kg commercial phase II compost (CNC). Each hybrid had two replicates (trays). The cultivation conditions were the same as described in a previous study [24]. Trays were distributed randomly on five shelf-layers in the growing room.

### Quantification of bruising sensitivity (BS)

Mushrooms of each hybrid were picked at their respective peaks of the first and second flush for screening of bruising sensitivity (BS). BS measurements were performed following the protocol described by Weijn et al (2012). In short, mushrooms were bruised mechanically and pictures were taken of the mushrooms after incubation for 60 minutes at room temperature in a humid chamber. BS was quantified from the pictures with computer image software, using the CIE *L* ^∗^ *a* ^∗^ *b* ^∗^ color system [24]. The bruising parameters used in this research are the whiteness index (WI) and the whiteness index difference (WI_DIFF). WI is calculated as *L* − (3 × b), as defined by Hunter (Hunterlab application note, 2008). The WI difference (BS) is the difference between the average WI of the bruised area and the average WI of the control (non-bruised) area, and the values for WI_DIFF differ thus from 0 (no visible bruising) to higher values with increasing bruising sensitivity. For each hybrid 20 mushrooms from two replicates (ten per tray) were analyzed. In addition, the pictures were ranked by eye in order to check the correlation between the data scored using the computer and visual scoring.

### Combining ability of homokaryotic parental lines

Because of incompatibility of some parental lines, only a partial diallel design was available for the combining ability study envisaged. The performance data of the resulting F1 hybrids were analyzed in a way derived from Griffing’s method, i.e. only including one set of F_1_ hybrids but neither parents nor reciprocal F_1_ hybrids [5,26]. Since each homokaryotic parental line contributes half of the genetic information to the hybrid, the mean performance (MP) of a particular homokaryotic parent is half of the mean BS of all crossings having this line as one parent. The general combining ability (GCA) of a particular line was calculated as the deviation of the mean performance of the line from half of the overall mean of all crosses in the diallel matrix. The deviation of BS of a particular cross from its expectation was used as an estimate of the specific combining ability (SCA) of the hybrid. The representation of the mathematical relationship of each cross is:

$$\begin{array}{c} MP_{A}=T_{A}/2n_{A};MP_{B}=T_{B}/2n_{B}\\ G_{A}=MP_{A}-\left[\Sigma T/2N\right]\\ G_{B}=MP_{B}-\left[\Sigma T/2N\right]\\ E\left(X_{AB}\right)=\left[\Sigma T/N\right]+G_{A}+G_{B}=MP_{A}+MPb_{B}\\ S_{AB}=X_{AB}-E\left(X_{AB}\right) \end{array}$$

*∑T* is the total BS value of all the crosses in the diallel design, and *N* is the total number of crosses. *T*_*A*_ and *n*_*A*_ are the sum of BS values and number of crosses having line A as a parent. *T*_*B*_ and *n*_*B*_ is the sum of BS values and number of crosses having line B as a parent. *G*_A_ and *G*_B_ represent the GCA of lines A and B, respectively, and *S*_AB_ the SCA of hybrid A × B. *X*_AB_ is the BS value of hybrid A × B, and E(*X*_*AB*_) is the expected BS value of hybrid A × B.

Analyses of variance (ANOVAs) were done using the model *Y*= μ + *G* + ε to test the presence of significant differences between hybrids (G) within flushes, and the model *Y*= μ + *G* + *F* + *G*×*F* + ε to test also for the presence of significant flush (F), genotype (G) and genotype by flush interaction effects (G×F); *Y* represents the bruising sensitivity of hybrids, *G* the genotypic effect, F is the flush effect, *G* ×*F* is the genotype by flush interaction effect, and ε represents the error effect. The broad-sense heritability (*H* ^*2*^) for hybrid means was calculated for each flush by H^2^=*σ* ^2^_*G*_/(*σ* ^2^_*G*_ +*σ* ^2^ *e*/*n*), and across flushes by H^2^=*σ* ^2^_*G*_/[*σ* ^2^_*G*_ + (*σ* ^2^ *GF*/*r*)+ (*σ* ^2^ *e*/*nr*)], where *σ* ^2^_*G*_ represents the genetic variance, *σ* ^2^_*e*_ is the error variance, *σ* ^2^_*GF*_ was the interaction variance of genotype by flush, *n* was the number of replicates (*n*=2), and *r* was the flush number (*r*=2). Multiple comparison for all hybrids was based on Fisher’s unprotected LSD test (*P<0.05*).

## Results

### Recovery of homokaryons

All 18 strains mentioned in Table 1 were used to recover homokaryons. Previous research has shown that homokaryotic and heterokaryotic protoclones show in general considerable differences in colony morphology and growth rate. Mycelia of homokaryons grow generally slower than that of heterokaryons and show fewer branches when inspected by microscope. Putative homokaryons were selected using these criteria. The presence of only one single nuclear type in such protoclones was confirmed by the absence of size polymorphisms upon PCR with primers that generate DNA fragments of different sizes in heterokaryons (results not shown). Protoplasting succeeded for all the 18 strains, but not all constituent nuclei of each selected strain were recovered as homokaryons. Out of seven strains (TW1, CH1, WW1, WW7, TW7, CH2, and WW2) both homokaryotic parental types were recovered and of six strains (TW8, WB2, WB4, WB5, TW6 and TW5) only one homokaryotic parental type were recovered (Table 3). The constituent nuclei of the remaining five strains were not recovered. The recovery of homokaryotic parental lines is likely influenced by the regeneration ability of protoclones, i.e., either a nuclear type is not viable without its counterpart or its regeneration is so slow that these protoclones are overgrown by protoclones from the counterpart. Homokaryons of the same nuclear type showed generally homogeneous growth, and only one was selected and used for the diallel crosses.

**Table 3:** List of strains from which homokaryons were recovered.

No.	Name	sensitivity	Cap color	Homokaryon I	Homokaryon II
1	TW8	resistant	white	M31	
2	TW1	resistant	white	K2	K20
3	CH1	resistant	white	O1	O13
4	WW1	resistant	off-white	Mes09199	Mes09200
5	WB2	resistant	brown	Mes09143	
6	WB4	resistant	brown	Mes01557P8	
7	WB5	resistant	brown	S3	
8	WW7	sensitive	off-white	Z6	Z8
9	TW7	sensitive	white	Q1	Q26
10	TW6	sensitive	white	N8	
11	WB15	sensitive	brown	Mes09119	
12	CH2	resistant	white	CH2A	CH2B
13	WW2	unknown	white	Mes09206	Mes09208

**Figure 1:**
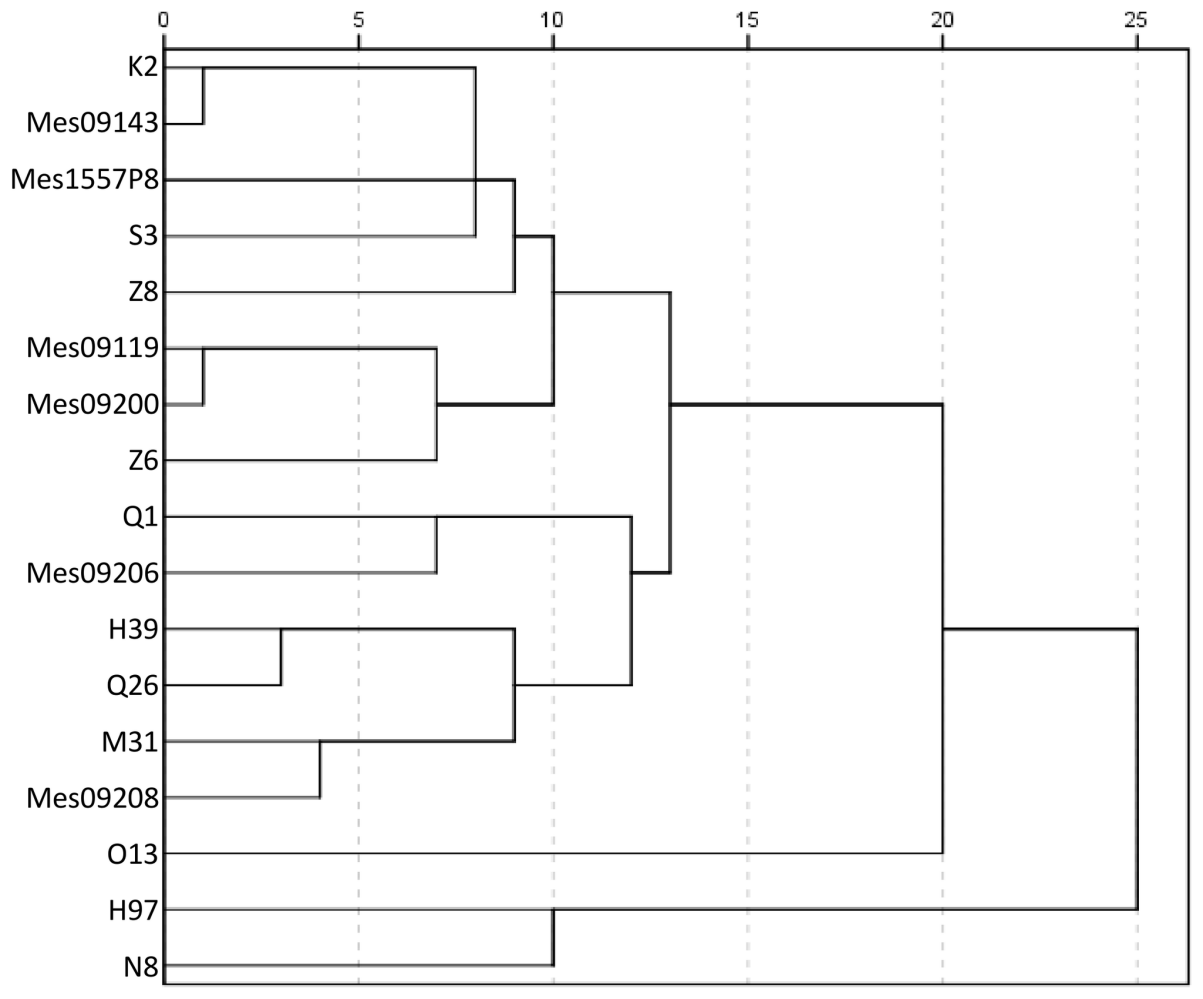
Genetic similarity of recovered homokaryons. The dendrogram was generated in SPSS with method of average linkage between groups. The scale bar at the top of the dendrogram shows the genetic distance between strains, the shorter the distance the higher the similarity.

The genetic diversity among homokaryotic lines was estimated with 535 SNP markers developed from the sequences of CH2B (JGI) and CH2A (Service Xs, Leiden). Although these SNP markers were selected on differences of only two lines, they give some indication of genetic relationship between the homokaryons used (Figure 1). The genetic distance between homokaryons recovered from the same commercial line was large and due to the fact that these commercial lines (CH lines) result from a cross between haploid single spore cultures of two genetically distinct traditional lines. Mes09199 and O1 were not included in the dendrogram because of insufficient marker data, and K20 was not included because of a very slow mycelium growth. Although SNP data derived from only two lines cannot be used to show pedigree relationship of the lines used, the data show at least that there is considerable genetic variation in the homokaryons used.

### Crossing and fruiting test

Nineteen recovered homokaryotic lines were intercrossed using a diallel crossing scheme (Table 4). The homokaryon K20 recovered from TW1 was not used for crossing because of its very slow growth. Not all combinations of the 19 lines were compatible. For instance, Mes01557P8 and Mes09200 were only compatible with Mes09143 and Z6. In contrast, Mes09143 was compatible with most parental lines apart from S3, Mes09199, Q1 and K2. The mating system of most basidiomycetes allows successful crossings among more than 90% of non-related individuals within a basidiomycete species [27,28]. In this study, however, the success rate of the crossings (less than 30%) was much lower, which might indicate a high frequency of one or more mating type alleles in the set of homokaryons recovered from genetically related heterokaryons. Fifty-one confirmed hybrids were finally obtained for fruiting and the assessment of bruising sensitivity. Forty-nine out of 51 hybrids produced mushrooms (Table 5). Hybrid 41 (O13 × N8) and 43 (Q26 × N8) failed to produce mushrooms. Some hybrids had a very low productivity in one tray or both trays (flush 2), which did not produce enough mushrooms to allow BS analysis.

**Table 4:** Compatibility between homokaryons used in a diallel crossing scheme.

9143	S3	1557P8	9200	9208	9199	9119	Q1	CH2A	N8	K2	Z6	CH2B	Q26	O13	9206	O1	M31	Z8	
*	I	**C**	**C**	**C**	I	**C**	I	**C**	**C**	I	**C**	**C**	**C**	**C**	**C**	**C**	**C**	**C**	9143
	*	I	I	I	I	I	I	I	I	I	I	**C**	I	I	I	**C**	I	I	S3
		*	I	I	I	I	I	I	I	I	**C**	I	I	I	I	I	I	I	1557P8
			*	I	*	I	I	I	I	I	**C**	I	I	I	I	I	I	I	9200
				*	I	I	I	I	I	I	**C**	I	I	I	*	I	I	I	9208
					*	I	I	I	I	**C**	**C**	I	I	I	I	**C**	I	**C**	9199
						*	I	I	I	I	I	**C**	I	I	I	I	I	I	9119
							*	I	I	I	I	**C**	*	**C**	I	I	I	I	Q1
								*	**C**	I	I	*	**C**	I	I	I	I	**C**	CH2A
									*	I	**C**	I	**C**	**C**	I	**C**	**C**	I	N8
										*	**C**	I	I	I	I	I	I	I	K2
											*	**C**	**C**	**C**	I	I	I	*	Z6
												*	**C**	I	I	**C**	I	**C**	CH2B
													*	**C**	I	**C**	**C**	I	Q26
														*	I	*	**C**	**C**	O13
															*	**C**	**C**	I	9206
																*	I	**C**	O1
																	*	**C**	M31
																		*	Z8

The names of lines starting with “Mes0” were trimmed to shorten them. Bold boxes with “C”: successful crosses; boxes with “I”: incompatible pairs; boxes with “*”: non-crossed pairs (same lines or lines from same parents).

**Table 5:** Summary of bruising sensitivity, cap color of hybrids in two successive flushes.

Hybrid	Parent 1	Parent 2	BS-flush 1 Mean (SD)	BS-flush 2 Mean (SD)	CC-flush 1 Mean (SD)	CC-flush 2 Mean (SD)
1	CH2A	Z8	37.23 (8.57)	32.63 (6.69)	62.58 (3.58)	63.26 (4.21)
2	CH2A	N8	26.35 (4.66)	16.58 (4.1)	66.01 (3.38)	66.95 (3.87)
3	CH2A	Mes09143	2.85 (3.02)	*	34.06 (5.21)	*
4	CH2A	Q26	35.13 (6.33)	23.87 (4.14)	65.13 (3.11)	71.70 (1.66)
5	CH2B	Z8	45.70 (3.96)	43.42 (7.87)	64.73 (2.38)	64.53 (3.91)
6	CH2B	O1	38.69 (1.82)	*	63.49 (2.25)	*
7	CH2B	Mes09143	8.21 (3.85)	7.48 (5.14)	34.61 (6.72)	36.6 (8.76)
8	CH2B	Q1	24.60 (4.97)	25.93 (3.84)	70.03 (2.67)	66.74 (4.04)
9	CH2B	Z6	34.88 (4.83)	29.94 (6.98)	57.75 (3.80)	61.11 (3.7)
10	CH2B	Q26	32.13 (3.14)	26.46 (6.39)	62.89 (4.42)	69.08 (2.67)
11	CH2B	Mes09119	37.97 (4.23)	35.23 (5.14)	65.86 (3.47)	67.02 (3.91)
12	CH2B	S3	6.84 (3.26)	9.39 (4.85)	14.37 (5.66)	14.00 (5.52)
13	K2	Z6	30.57 (4.70)	33.38 (4.01)	54.74 (4.91)	57.91 (3.30)
14	K2	Mes09199	17.65 (6.34)	14.56 (4.44)	28.71 (5.90)	43.45 (6.62)
15	M31	O13	29.48 (6.02)	30.66 (5.79)	61.13 (4.62)	67.97 (2.87)
16	M31	Z8	53.81 (4.58)	45.71 (6.33)	63.46 (3.04)	61.81 (3.69)
17	M31	Mes09143	3.01 (3.88)	6.25 (4.63)	28.44 (7.78)	32.6 (8.72)
18	M31	Q26	30.59 (7.09)	*	56.49 (2.57)	*
19	M31	Mes09206	30.12 (3.85)	35.68 (5.85)	55.90 (3.04)	59.99 (5.22)
20	Mes09199	Z8	26.71 (6.75)	13.26 (3.93)	34.29 (6.31)	44.38 (4.25)
21	Mes09199	Z6	14.64 (6.53)	12.37 (5.29)	27.27 (5.68)	44.03 (8.26)
22	Mes09200	Mes09143	3.09 (3.24)	6.75 (4.07)	28.87 (4.86)	38.77 (7.57)
23	Mes09200	Z6	21.40 (4.20)	16.92 (3.11)	45.46 (5.46)	50.55 (5.19)
24	Mes09143	Z6	10.84 (4.27)	11.44 (5.26)	18.30 (4.56)	34.86 (6.89)
25	Mes09143	Z8	9.07 (4.43)	2.92 (3.66)	33.39 (6.56)	31.01 (10.91)
26	Mes09143	Q26	6.62 (4.31)	2.63 (2.57)	31.18 (5.23)	29.27 (4.94)
27	Mes09143	Mes09119	3.78 (3.85)	6.11 (5.36)	28.14 (9.56)	29.1 (7.79)
28	Mes09143	Mes09206	3.28 (3.86)	8.46 (5.04)	21.28 (7.00)	31.71 (6.89)
29	Mes09143	Mes09208	5.99 (3.83)	3.65 (3.90)	37.05 (12.58)	54.92 (7.25)
30	O1	S3	5.82 (5.09)	8.6 (4.18)	22.90 (10.08)	3.55 (3.97)
31	O1	Z8	48.66 (4.66)	36.29 (5.28)	64.92 (2.87)	58.98 (4.58)
32	O1	Mes09143	14.81 (5.23)	*	41.74 (5.14)	*
33	O1	Q26	24.73 (4.55)	*	63.23 (3.44)	*
34	O1	N8	32.51 (8.12)	20.3 (4.30)	61.28 (2.88)	59.37 (3.16)
35	O1	Mes09206	33.48 (4.55)	22.08 (3.70)	60.95 (3.29)	63.69 (3.49)
36	O1	Mes09199	29.93 (6.76)	16.62 (3.84)	34.75 (4.54)	47.17 (5.21)
37	O13	Q1	20.70 (5.10)	12.27 (4.38)	63.40 (5.19)	60.06 (3.15)
38	O13	Z6	35.54 (5.71)	23.56 (5.82)	52.02 (8.19)	60.79 (3.33)
39	O13	Z8	57.01 (6.72)	29.29 (4.01)	58.08 (3.93)	59.4 (3.75)
40	O13	Q26	26.33 (3.37)	16.85 (3.10)	63.79 (5.09)	67.73 (2.84)
41	O13	N8	-	-	-	-
42	O13	Mes09143	2.95 (2.72)	3.65 (4.05)	22.31 (4.99)	33.06 (7.67)
42	Q26	N8	-	-	-	-
44	Z6	Mes09208	18.23 (4.19)	27.89 (1.97)	63.02 (1.69)	58.81 (1.83)
45	Z6	Q26	38.01 (6.57)	27.77 (7.21)	61.55 (3.73)	65.23 (2.28)
46	Z6	N8	35.42 (3.64)	25.78 (4.81)	54.51 (4.57)	59.64 (3.67)
47	M31	N8	31.76 (3.75)	27.94 (5.66)	63.77 (1.47)	64.03 (2.65)
48	Mes09143	N8	4.41 (3.62)	5.11 (3.59)	27.37 (3.62)	39.05 (4.30)
49	Mes01557P8	Mes09143	3.61 (4.08)	7.51 (4.62)	11.29 (6.59)	16.16 (9.39)
50	Mes01557P8	Z6	11.7 (5.26)	13.62 (6.97)	19.96 (10.07)	21.08 (9.40)
51	CH2A	CH2B	15.58 (3.46)	14.59 (5.01)	70.56 (2.70)	69.03 (3.25)

“* ” indicates hybrids did not produce enough mushrooms to allow BS and CC analysis; “- ” indicates hybrids did not produce mushrooms at all. The higher BS value indicates higher bruising sensitivity, and the higher CC value indicates whiter mushroom cap. SD indicates the standard deviation.

### Variation in bruising sensitivity (BS) among hybrids

**Figure 2:**
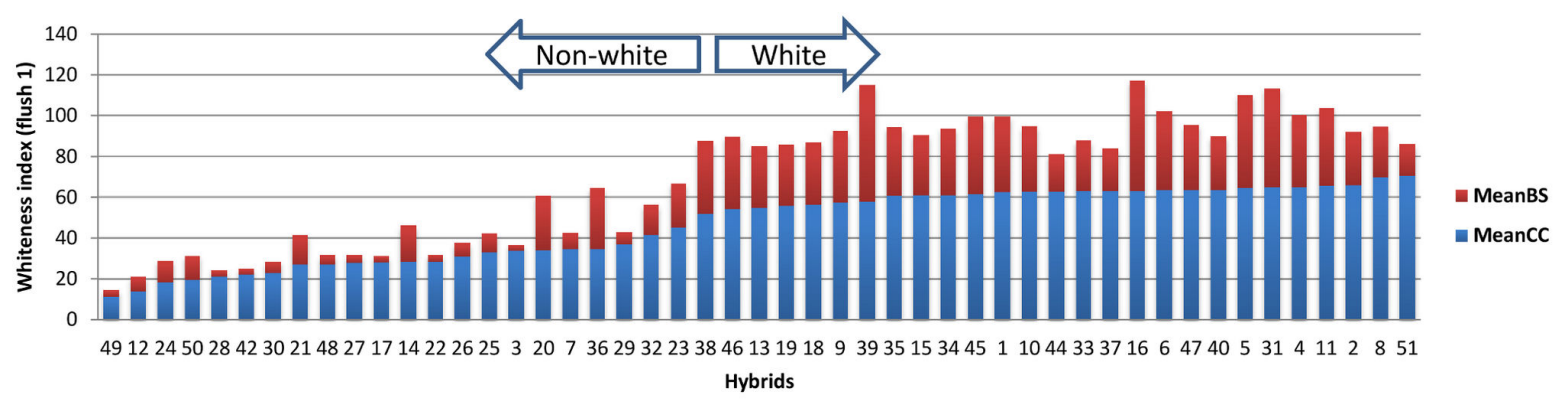
Bruising sensitivity (BS) and cap color (CC) of hybrids in flush 1. The hybrids were sorted by the mean CC (whiteness index of cap color). The BS and CC values are plotted on top of each other for each hybrid.

The test for BS (expressed as whiteness index) showed a large variation among hybrids, which ranged from 2.85 to 57.01 in flush 1 and from 2.63 to 45.71 in flush 2 (Table 5). The higher the value the more the level of discoloration is. A good correlation was found between data of computer software and visual scoring. The BS of hybrids in flush 1 was highly correlated with that in flush 2 (*r* = 0.82; Pearson, *P*<0.001). ANOVA showed that genotype and flush were both significant factors for BS, and interaction effect of genotype by flush was significant as well (*P*<0.05). Mushrooms of flush 2 were significantly less sensitive than mushrooms of flush 1 (*P*<0.05). The cap color (CC) was also measured with the same imaging system, which ranged from 11.29 to 70.56 in flush 1 and from 3.55 to 71.70 in flush 2 (Table 5). The higher the value the whiter the mushroom is. The overall mean of CC-flush 2 was significantly higher than the overall mean of CC-flush 1 (P<0.05). Non-white hybrids (including off-white, light brown and brown hybrids) were generally less sensitive than white hybrids in both flushes, which is consistent with the findings of a previous study [24]. For instance, the bar chart in Figure 2 shows that white mushrooms of flush 1 have generally higher BS than non-white hybrids.

Bruising sensitivity is a quantitative trait and shows continuous variation among strains. Broad-sense heritability (*H* ^*2*^) of BS-flush 1 and BS-flush 2 were 0.99 and 0.97 respectively, and also high across flushes (H^2^ = 0.93). This indicates that the genotypic variation for BS is highly inheritable.

### General combining ability

The diallel crossing scheme among homokaryons allowed testing each homokaryon for BS in different genetic backgrounds. The mean performance of parental lines in flush 1, flush 2 and the two flushes combined are shown in Table 6. It turned out that not all homokaryotic lines derived from resistant strains gave rise to bruising resistant hybrids. This holds true, for instance of crosses with M31, CH2B, O1 and O13, homokaryons derived from resistance strains. Among all these tested parental lines Mes09143 was the most resistant line, and Z8 the most sensitive one.

**Table 6:** Mean performance of parental lines in crosses for BS and the corresponding GCA estimates based on data from flush 1 & 2 and combined analyses.

	Number of hybrids			Mean performance			GCA		
Parent	Flush 1	Flush 2	Combined	Flush 1	Flush 2	across flush	Flush 1	Flush 2	across flush
Mes09143	27	23	27	2.89	3.07	3.08	-8.57	-6.49	-7.5
S3	3	4	4	3.25	4.5	4.07	-8.21	-5.06	-6.51
Mes01557P8	4	4	4	3.81	5.25	4.53	-7.65	-4.31	-6.05
Mes09200	4	4	4	6.13	5.92	6.02	-5.33	-3.64	-4.56
Mes09208	3	3	3	5.04	5.86	5.45	-6.42	-3.7	-5.13
Mes09199	8	8	8	11.16	7.11	9.14	-0.3	-2.45	-1.44
Mes09119	4	4	4	10.44	10.34	10.39	-1.02	0.78	-0.19
Q1	4	4	4	11.33	9.55	10.44	-0.13	-0.01	-0.14
CH2A	9	8	10	11.08	10.95	10.11	-0.38	1.39	-0.47
N8	10	10	10	13.06	9.57	11.32	1.6	0.01	0.74
K2	4	4	4	12.03	12	12.01	0.57	2.44	1.43
Z6	19	19	19	12.73	10.98	11.86	1.27	1.42	1.28
CH2B	17	16	17	13.26	12.02	12.86	1.8	2.46	2.28
Q26	13	10	14	13.56	9.75	12.18	2.1	0.19	1.6
O13	11	12	12	14.29	9.66	12.03	2.83	0.1	1.45
Mes09206	6	6	6	11.12	11.09	11.11	-0.34	1.53	0.53
O1	14	10	15	14.79	10.4	12.49	3.33	0.84	1.91
M31	11	10	12	14.92	14.64	14.87	3.46	5.08	4.29
Z8	13	13	13	21.05	15.53	18.29	9.59	5.97	7.71

N is the number of trays having the homokaryon as a parent (two replicates per hybrid), odd number indicates one tray of the hybrid did not produce enough mushrooms; MP is the mean performance of individual homokaryons (the mean BS of hybrids); GCA is the general combining ability; negative GCA indicates lower bruising sensitivity positive GCA indicates higher bruising sensitivity compare to the general mean BS of all hybrids in the diallel matrix.

**Figure 3:**
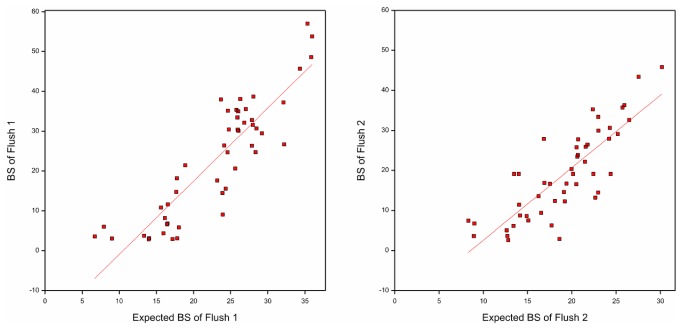
Correlation of observed BS of hybrids and their expected value (flushes 1 and 2).

The expected and observed performance of hybrids for BS in flushes 1 and 2 are depicted in Figure 3. The actual performance showed a clear positive correlation with the expectation. The correlation coefficients for the respective flushes were 0.88 and 0.86 (*P*<0.001) indicating that the variation in BS among hybrids has an additive, possibly polygenic nature. Under polygenic inheritance with additive gene action offspring resemble more closely the average of their parents (the mid parent) than either one of the individual parents [29].

**Figure 4:**
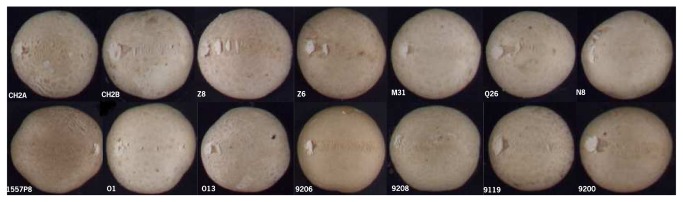
Bruised mushrooms of hybrids with Mes09143 as one of the parents (flush 1, 60 min after bruising).

The GCA of a homokaryon for BS is equivalent to its breeding value [30]. BS is defined as the discoloration relative to the non-bruised area and is zero with no visible bruising damage and its value increases with increasing discoloration. A positive value of GCA indicates thus a higher BS than the average and a negative value the opposite, i.e. a lower BS than the average. The GCA analysis shows that the parental homokaryons Mes09143, S3, Mes01557P8, Mes09200 and Mes09208 have a high negative GCA, and are thus good donors for bruising resistance (BR) (Table 6). Especially Mes09143 is of interest with negative GCA values of -8.57 in flush 1 and -6.49 in flush 2. Its GCA estimate was based on the highest number of crosses due to its rare mating type and is thus rather reliable. The best performing homokaryon Mes09143, derived from a wild resistant brown strain, showed no or hardly any discoloration of mushrooms in all crosses (Figure 4). The mean performance of crosses with Mes09143 was 3.08 across flushes, and the individual hybrids with this line in their pedigree varied in BS between 2.63 and 14.81. This indicates that Mes09143 is carrying dominant alleles for BR and an attractive donor for BR.

**Figure 5:**
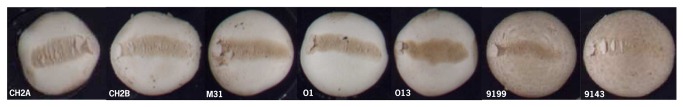
Bruised mushrooms of hybrids with Z8 as one of the parents (flush 1, 60 min after bruising).

At the opposite end of the spectrum, Z8 is a good donor for BS, as Z8 showed in the two successive flushes GCA values of 9.59 and 5.97 respectively. Z8 and Z6 are the two constituent homokaryons of a white wild strain sensitive to bruising (WW7). All hybrids with Z8 as a parental line were sensitive to bruising except for the one with Mes09143 (the strain that was dominant for BR) (Figure 5). The mean performance of Z8 across flushes was 18.29, and the individual hybrids with this line as a parent varied in BS across flushes between 5.99 and 49.87. This indicates that Z8 presents a dominant pattern for bruising sensitivity (BS). Z6 did not show a clear pattern in the variation of BS between hybrids.

**Figure 6:**
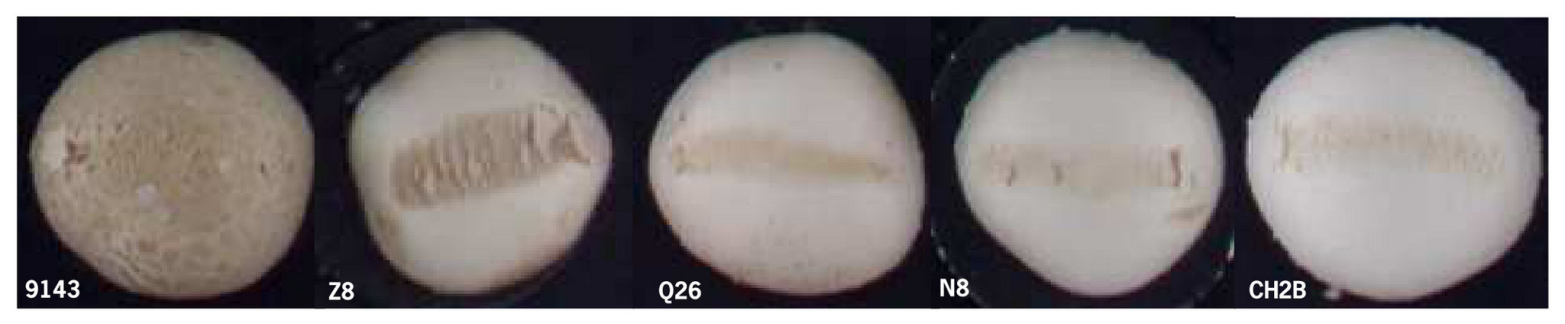
Bruised mushrooms of hybrids with CH2A as a parental line (flush 1, 60 min after bruising).

**Figure 7:**
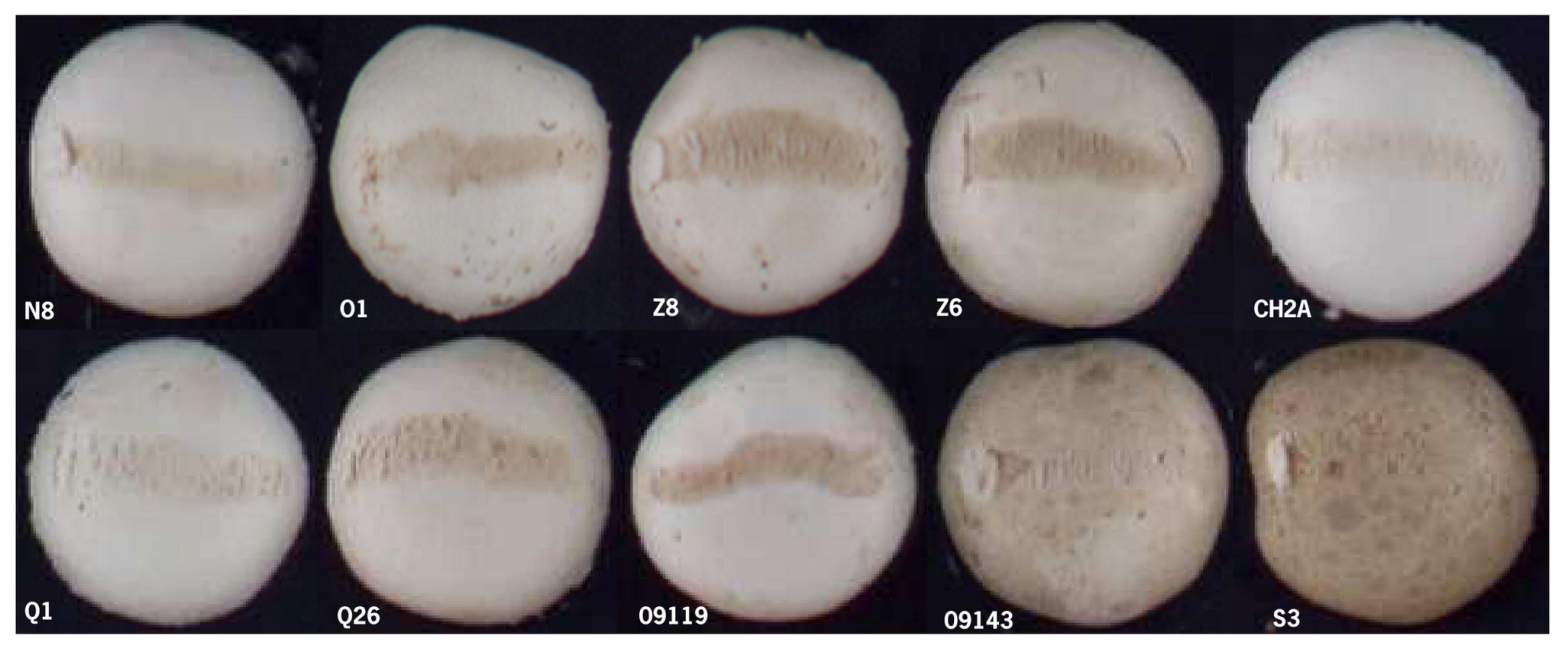
Bruised mushrooms of hybrids with CH2B as a parental line (flush1, 60 min after bruising).

Some lines did show intermediate GCA estimates, e.g. Mes09206, Q1, CH2A, N8, K2, and Z6, CH2B, nevertheless, derived from insensitive strains. For instance, hybrids with either CH2A or CH2B as a parent varied considerably in sensitivity, depending on the partner in the hybrid (Figures 6 & 7). The mean performance of CH2A in crosses was 10.11 across flushes, and the BS of its individual hybrids across flushes varied between 2.85 and 34.93; the mean performance of CH2B across flushes was 12.86, and the BS of its individual hybrids across flushes varied between 7.84 and 44.53. The cross between CH2A and CH2B represents the most bruising-resistant commercial white hybrid (CH2) currently on the market. This suggested that its parental homokaryons complement each other genetically resulting in a hybrid with a relative resistance to bruising, but genes involved are not dominant.

### Specific combining ability

The deviation of BS from the expected value was used as a measure of SCA. A positive value for SCA indicates that the BS of the cross is higher than expected (more sensitive), and negative SCA indicates the BS value of the cross is lower than expected (more resistant). The SCA values of all hybrids in flushes 1 and 2 are presented in Table 7. All brown hybrids had negative SCA except for the cross Mes09200×Z6. As mentioned previously, Mes09200 was a homokaryon recovered from an off-white stain but expressed a brown cap color in all crosses and was thus an exceptional crossing partner. All, except one, homokaryons derived from brown varieties gave rise to bruising resistant hybrids and homokaryons from white varieties resulted in either sensitive or resistant crosses. It is remarkable that most crosses among ‘white’ homokaryons were more sensitive than expected. Nevertheless, there is still substantial variation in bruising sensitivity among white crosses. The white crosses of CH2A×CH2B, O13×Q1 and O13×Q26 were more resistant than expected (more negative SCA than expected), in which the cross of CH2A×CH2B was the most insensitive one among white hybrids. The homokaryons (parental lines) of crosses mentioned above were all derived from white commercial varieties, and these varieties were genetically related. Combinations between these homokaryons are thus a special case and our data indicate that these lines complement each other genetically resulting in a relatively low BS.

**Table 7:** SCA of hybrids in two successive flushes.

Hybrid	Parent 1	Parent 2	SCA (flush 1)	SCA (flush 2)		Hybrid	Parent 1	Parent 2	SCA (flush 1)	SCA (flush 2)
1	CH2A	Z8	5.10	6.15		26	Mes09143	Q26	-9.83	-10.19
2	CH2A	N8	2.21	-3.94		27	Mes09143	Mes09119	-9.55	-7.30
3	CH2A	Mes09143	-11.12	-		28	Mes09143	Mes09206	-10.73	-5.70
4	CH2A	Q26	10.49	3.17		29	Mes09143	Mes09208	-1.94	-5.28
5	CH2B	Z8	11.39	15.87		30	O1	S3	-12.22	-6.30
6	CH2B	O1	10.64	-		31	O1	Z8	12.82	10.36
7	CH2B	Mes09143	-7.94	-7.61		32	O1	Mes09143	-2.87	-
8	CH2B	Q1	0.01	4.36		33	O1	Q26	-3.62	-
9	CH2B	Z6	8.89	6.94		34	O1	N8	4.66	0.33
10	CH2B	Q26	5.31	4.69		35	O1	Mes09206	7.57	0.59
11	CH2B	Mes09119	14.27	12.87		36	O1	Mes09199	3.98	-0.89
12	CH2B	S3	-9.67	-7.13		37	O13	Q1	-3.89	-6.94
13	K2	Z6	5.81	10.40		38	O13	Z6	9.55	2.92
14	K2	Mes09199	-5.54	-4.55		39	O13	Z8	22.70	4.10
15	M31	O13	1.30	6.36		40	O13	Q26	-0.49	-2.56
16	M31	Z8	17.84	15.54		42	O13	Mes09143	-13.20	-9.08
17	M31	Mes09143	-14.80	-11.46		44	Z6	Mes09208	0.46	11.05
18	M31	Q26	2.11	-		45	Z6	Q26	11.72	7.04
19	M31	Mes09206	4.08	9.95		46	Z6	N8	9.63	5.23
20	Mes09199	Z8	-5.50	-9.38		47	M31	N8	3.78	3.73
21	Mes09199	Z6	-9.25	-5.72		48	Mes09143	N8	-11.54	-7.53
22	Mes09200	Mes09143	-5.93	-2.25		49	Mes01557P8	Mes09143	-3.09	-0.81
23	Mes09200	Z6	2.54	0.02		50	Mes01557P8	Z6	-4.84	-2.61
24	Mes09143	Z6	-4.78	-2.61		51	CH2A	CH2B	-8.76	-8.38
25	Mes09143	Z8	-14.87	-15.68						

A positive SCA indicates that the BS of the cross is higher than expected (more sensitive), and negative SCA indicates the BS value of the cross is lower than expected (more resistant). “- ” indicates missing data because of no mushrooms or not enough mushrooms.

## Discussion

In this study, the GCA of a particular line is calculated as the deviation of the mean performance of a particular line from half of the overall mean of all crosses in the diallel scheme. GCA is equivalent to the breeding value of an individual [30], and the GCA of a line is a reflection of the number of loci with positive alleles for the trait of interest and their effect [31]. The high negative GCA of Mes09143 indicates that this strain has a high number of loci with alleles having a favorable effect on the level of bruising resistance (BR). Therefore such a strain can be an interesting source (donor) for genetic improvement of bruising sensitive mushrooms. The opposite accounts for Z8, probably having a low frequency for genes with a beneficial effect on BR and thus a good combiner for BS in the segregation analysis. The ones with intermediate GCA are neither donors for BR nor donors for BS, because the trait expression in their crosses is always determined by the counter parents. They might be good recipients in breeding programs if they carry other favorable agronomic traits. GCA analysis is thus useful to select parental lines for different purposes. Parental lines with a favorable GCA can be used to stack positive alleles of genes involved in the trait of interest through recombination and selection, whereas parental lines with opposite GCA values are good parents for the creation of a dedicated mapping population to study the genetics of traits of interest.

Negative SCA estimates were observed in brown hybrids and positive SCA estimates in most white hybrids. This might indicate that the cap color interferes with bruising resistance. Whether the major gene for cap color has a pleiotropic effect and thus also influences BS or other genes linked to this gene are involved is not known, which can be seen from a segregation analysis. This also indicates that breeders should not only use white lines in further breeding programs to improve bruising resistance, but also brown lines. Not only the sign of SCA value, also the magnitude of the value is meaningful. It was suggested that the high magnitude means high genetic divergence and high heterosis in the hybrids [32]. In addition, hybrids displaying heterosis are said to have favorable combining ability [13]. The lowest negative SCA estimate of hybrid strain CH2 among all the white hybrids indicates that heterosis due to genetic complementation is an important cause for its relatively low sensitivity to bruising. Similarly, heterosis might be an important factor explaining the bruising resistance of hybrids between the most bruising resistant homokaryon Mes09143 and the most bruising sensitive homokaryon Z8. The difference between the observed performance of an F1 hybrid and the expected value based on the GCA of its parents indicate gene interactions [8]. The better than expected performance of the hybrid between Mes09143 and Z8 cannot be explained by additive effects alone but indicate interference between alleles (dominance or even over-dominance). These lines widely differing in the breeding value for sensitivity to bruising, can be used to generate a segregating population for mapping purposes. In a successive project, we will generate a segregating population from this hybrid to map QTLs and find candidate genes involved in bruising sensitivity.

**Figure 8:**
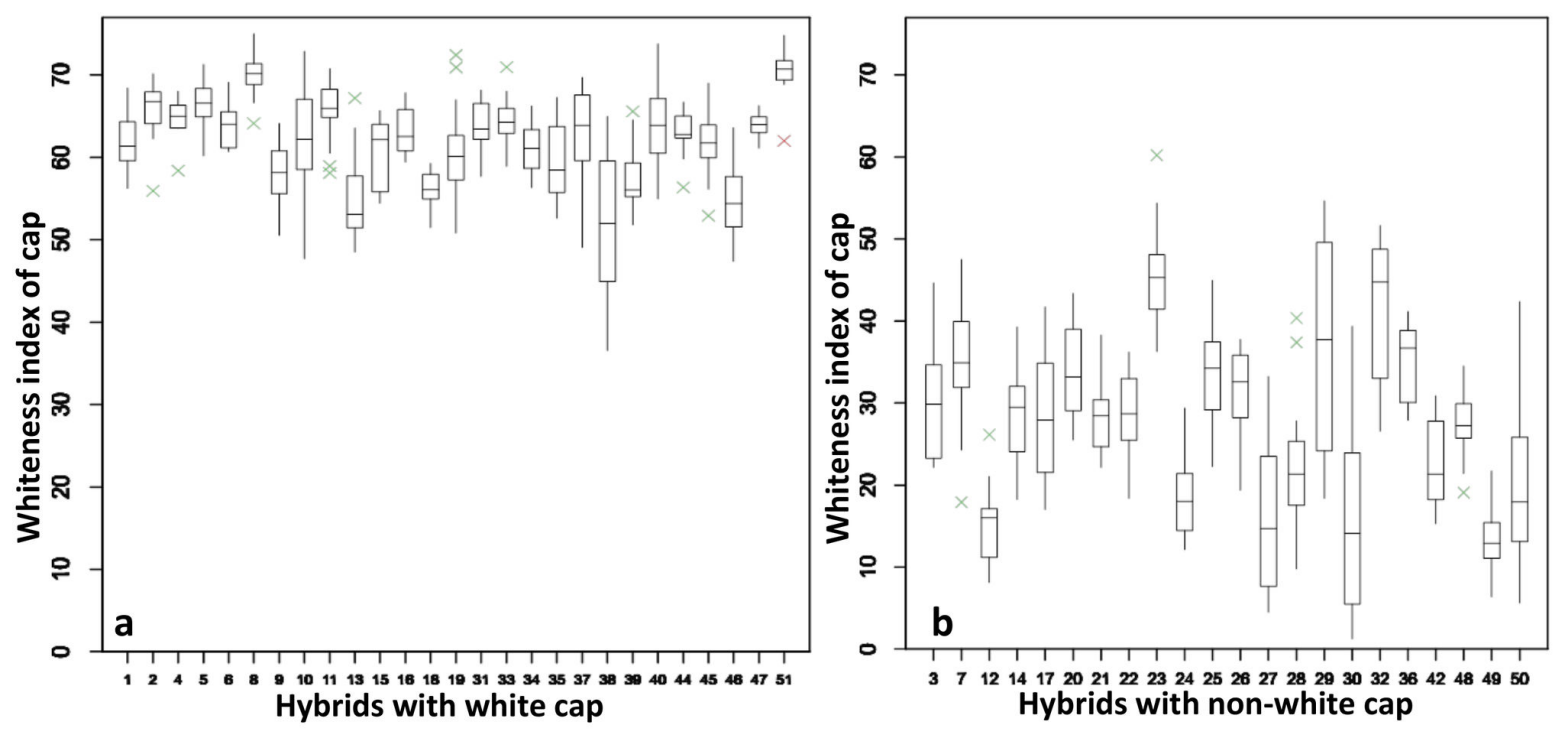
a. Boxplots describing variation in cap whiteness for each hybrid classified as white (flush 1). he box spans the interquartile range of the values of cap whiteness of each hybrid, so that the middle 50% of the data lie within the box, with a line indicating the median. Whiskers extend beyond the ends of the box as far as the minimum and maximum values. The crosses indicate outliers. The cap whiteness of these hybrids were measured based on 20 individual mushrooms of flush 1 per hybrid. Long boxes indicate large variation of cap whiteness, and short boxes indicate small variation of cap color. By comparing Figure 8a and Figure 8b individual mushrooms of non-white hybrids show larger difference of cap color than that of white hybrids. b. Boxplots describing variation in cap whiteness for each hybrid classified as non-white (flush 1).

Cap color of button mushrooms is controlled by a major QTL on chromosome 8 with a recessive allele for white [33]. Therefore it was not surprising to see two distinct groups of hybrids for cap color, i.e. white and non-white. The ones depicted in Figure 8a have a more or less white cap color with a mean whiteness index ranging from 56.29 (off-white) to 69.81 (white) in flush 1 (values are in Table 5). The box plot for cap color of these hybrids based on 20 individual mushrooms of flush 1 per hybrid shows little variation in cap color. The group of hybrids with a non-white cap color had in their pedigree at least one homokaryon (Mes09143, Mes01557P8, S3) from wild accessions with a brown cap color (Figure 8b). All these crosses produced mushrooms with cap colors varying from light brown to brown (and this range is designated as non-white) with a range in whiteness index of cap color from 13.66 (brown) to 48.07 (light brown) in flush 1. The larger variation of non-white cap color compared to white cap color indicates a more complex genetic base for cap color than just one gene. The variation might be due to the dosage variation of brown cap color QTL and the involvement of modifier genes. The modifier loci for cap color have been found in a previous study [33]. Hybrid 27 (Mes09143 × Mes09119), for example, with both parental lines recovered from brown heterokaryons produced mushrooms with brown caps. Hybrid 12 (CH2A/derived from a white hybrid × S3/derived from a brown hybrid) produced brown mushrooms as well. However, a cross (CH2B/derived from a white hybrid × Mes09119/derived from a brown hybrid) produced white mushrooms although Mes09119 was derived from a brown strain and CH2B from a white strain. This indicates that WB15, from which Mes09119 was obtained, is heterozygous for the major locus for cap color. Another exceptional strain was WW1, an off-white strain identified in a previous study. Both homokaryotic parental lines apparently have the brown QTL allele for cap color since all hybrids having one of them as parent were brown or light brown.

In addition to the variation in cap color between the non-white hybrids, also individual mushrooms of the same non-white hybrids show large differences with respect to cap color. Especially for crosses between homokaryons recovered from white and brown varieties, mushrooms are produced with high variation in cap color, varying from brown to light brown, i.e. hybrids 27, 29, 30, 32 (Figure 8b). Hybrids among brown strains (Figure 8b, hybrid 49) and among white strains (Figure 8a, hybrids 2, 4, 6 etc.) produced mushrooms with much less variation in color (thus either brown or white). We can therefore designate the crosses as brown, light brown, off-white, and white. This might indicate the incomplete expression of brown alleles.

The WI of control area for white hybrids is always higher owing to its light color than that of the bruised area; even very light discoloration can be seen. All brown hybrids used in this study were clearly less brown than present-day brown commercial strains and allowed good assessment of discoloration upon bruising. Nevertheless, a very light discoloration might be partly obscured by the background color despite the fact that the discoloration is assessed relatively to the non-bruised background color of the same mushroom. This might also be the reason why brown strains generally show less discoloration than white strains. Next to this, strong scaling of some non-white caps might also influence the BS measurement. The WI of the control area was sometimes lower than that of the bruised area, which gave a negative BS value. Occasionally the cap skin was damaged so severely that white tissue underneath the skin was visible. Such white tissue might interfere with the assessment of BS, although care was taken to select only areas with intact skin for the measurements. Nevertheless white spots maybe in some cases the reason for negative values of discoloration. Negative values were corrected into zero if there was really no discoloration.

Mushroom breeding is similar to plant breeding but differs in a number of aspects. Mushrooms have a relatively short life cycle with a haploid phase (homokaryon) that can be easily propagated and maintained as infertile vegetative mycelium. Homokaryons can be considered equivalent to double haploids or inbred lines used nowadays in breeding of many plant species. The nuclei of fertile diploid mycelium (heterokaryon) stay apart (n + n) and do not fuse as in diploid plants (2n). The constituent nuclei can thus be recovered from the heterokaryons as haploid homokaryons, and the intact and original genome combination can be maintained for ever. This is a practical advantage of mushroom breeding over plant breeding. A complication is that in button mushrooms the mating type of homokaryons (haploids) limits the production of heterokaryons. It is thus wise to start heterosis breeding with a broad selection of compatible lines in order to have sufficient lines to complete a full diallel scheme. The fact that this paper reports for the first time the estimation of breeding value by using diallel crossings underlines that mushroom breeding lacks behind plant breeding. However, it also shows that the same techniques and strategies used in plant breeding can be used in mushroom breeding.

This study was the first attempt to analyze the combining ability of the parental homokaryons of a set of button mushrooms hybrids. It has shown that in *A. bisporus* bruising sensitivity is a highly inheritable trait. The study also showed that this approach is an excellent way to estimate the breeding value of homokaryons of button mushroom, and facilitates the selection of parental lines for heterosis breeding. In a follow-up study, hybrids of lines differing strongly in bruising sensitivity will be used to generate segregating populations to enhance our knowledge of genetics of bruising sensitivity.
